# Immediate response of myocardium to pressure overload includes transient regulation of genes associated with mitochondrial bioenergetics and calcium availability

**DOI:** 10.1590/S1415-47572010005000004

**Published:** 2010-03-01

**Authors:** Ana Carolina Deckmann, Thaís Holz Theizen, Francisco Javier Medrano, Kleber Gomes Franchini, Gonçalo Amarante Guimarães Pereira

**Affiliations:** 1 Departamento de Genética e Evolução, Instituto de Biologia, Universidade Estadual de Campinas, Campinas, SPBrazil; 2 Departamento de Medicina Interna, Escola de Medicina, Universidade Estadual de Campinas, Campinas, SPBrazil

**Keywords:** pressure overload, myocardial hypertrophy, gene expression, SERCA2

## Abstract

Ventricular hypertrophy is one of the major myocardial responses to pressure overload (PO). Most studies on early myocardial response focus on the days or even weeks after induction of hypertrophic stimuli. Since mechanotransduction pathways are immediately activated in hearts undergoing increased work load, it is reasonable to infer that the myocardial gene program may be regulated in the first few hours. In the present study, we monitored the expression of some genes previously described in the context of myocardial hypertrophic growth by using the Northern blot technique, to estimate the mRNA content of selected genes in rat myocardium for the periods 1, 3, 6, 12 and 48 h after PO stimuli. Results revealed an immediate switch in the expression of genes encoding alpha and beta isoforms of myosin heavy chain, and up-regulation of the cardiac isoform of alpha actin. We also detected transitory gene regulation as the increase in mitochondrial cytochrome c oxidase 1 gene expression, parallel to down-regulation of genes encoding sarco(endo)plasmic reticulum Ca^+2^ ATPase and sodium-calcium exchanger. Taken together, these results indicate that initial myocardial responses to increased work load include alterations in the contractile properties of sarcomeres and transitory adjustment of mitochondrial bioenergetics and calcium availability.

Myocardial hypertrophy is associated with the high risk of cardiac mortality due to its established role in the development of cardiac failure, one of the leading causes of death world-wide [World Health Organization - 2007 Prevention of Cardiovascular Diseases: Guidelines for assessment and management of total cardiovascular risk.]. Myocardial hypertrophy is defined as an abnormal increase in heart muscle mass in response to exposure to neurohumoral factors and increased work load conditions. In both cases, the myocardium undergoes excessive growth as an adaptive response. Nevertheless, if sustained, this adaptive hypertrophy leads to dysfunctional and uncompensated hypertrophy, with heart failure as a possible outcome. For a comprehensive review of this subject, see Diamond and Phillips (2005).

Therefore, much attention has been given to investigating subcellular events underlying the cardiac hypertrophic response, in order to detect possible targets to prevent its progression to heart failure. A growing number of intracellular signaling pathways have been characterized, including specific G-protein isoforms, small GTPases, mitogen-activated protein kinase cascades, adhesion and cytoskeletal proteins, calcium-mediated signaling and microRNA expression (see review in Barry *et al,* 2008). These multiple signaling pathways lead to the regulation of gene expression, among which are the so-called IEGs (immediate expressed genes, as c-*jun*, c-*fos*, c-*myc*, *egr1*) and genes of the fetal program (beta isoform of myosin heavy chain, skeletal isoform of alpha-actin, atrial and brain natriuretic peptides) (Clerk *et al.*, 2007).

Furthermore, in an attempt to delineate the entire set of transcriptional responses of cardiac cells exposed to these multiple signaling pathways, high-scale techniques were employed to investigate the global gene expression involved in myocardial hypertrophy (Anisimov *et al.*, 2002; Weinberg *et al.*, 2003; Zhao *et al.*, 2004; Strom *et al.*, 2004; van den Bosch *et al.*, 2006). As a result, many genes were identified as having been regulated days or even weeks after stimuli in several models of cardiac hypertrophy, but much still remains to be investigated before this knowledge can be transformed into potential pharmacological therapies (Barry *et al.*, 2008).

One of the reasons why it is difficult to adapt gene expression results to effective therapeutics is the complexity of regulatory networks coupling gene expression to protein synthesis and, ultimately, to protein localization and activity. Various recent studies have shown that the regulation of myocardial gene expression is highly sophisticated, and includes multiphasic transcriptional response (Cullingford *et al.*, 2008), epigenetic mechanisms (McKinsey and Olson, 2004) and selective translation of transcribed mRNAs (Spruill *et al.*, 2008).

The challenge to deciphering gene regulatory networks may be facilitated if even discrete changes in transcriptional activity are reported and analyzed together with functional assays to check whether these gene alterations are really reflecting phenotypic changes, or are simply a part of signaling itself. Furthermore, the time window between stimuli input and cellular adaptation must be carefully observed, since, in samples collected days or weeks after stimuli, several intermediate adjustments of cellular physiology may be neglected (Glauser and Schlegel, 2006).

In this context, we report herein the transient regulation of various genes encoding essential components of cardiac cells, in the first few hours after induction of myocardial hypertrophy in rats. The expression of genes encoding alpha myosin heavy chain (α-MHC), beta myosin heavy chain (β-MHC), alpha cardiac actin (ACTC1), sarco(endo)plasmic reticulum Ca^+2^ ATPase (SERCA2), sodium-calcium exchanger (NCX) and cytochrome c oxidase 1 (COX1), were analyzed during a time span of 1, 3, 6, 12 and 48 h after transverse aortic constriction (TAC) in adult Wistar rats. Gene expression was studied by using Northern blot hybridization of mRNAs collected from TAC or sham-operated (control) rats to cDNA probes isolated from a commercial cDNA library (5' STRETCH PLUS – Clontech, USA). Detailed methods are presented as Supplementary Material.

As expected for the short time span investigated here, rats that underwent the TAC procedure showed no significant increase in the left ventricular weight (LV) / body weight (BW) ratio, despite a significant increase in the systolic pressure gradient (SPG) with respect to the controls. These data are summarized in Table 1.

According to gene expression results and specifically LV in TAC rats, there were alterations in the mRNA levels of genes encoding components of the contractile apparatus (α-MHC, β-MHC, ACTC1), calcium cycling (SERCA2 and NCX), and the mitochondrial electron chain (COX1). Northern blot assays and respective densitometric readings are shown in Figure 1.

The two isoforms of the myosin heavy chain showed inverse regulation with time (Figure 1A and 1B). Whereas in α*-MHC* there was a progressive down-regulation, more obvious at later periods (6 to 48 h), the β*-MH*C gene was progressively up-regulated after 1 h. Cardiac α-actin (*ACTC1*, Figure 1C) was strongly up-regulated 1 h after pressure overload, its expression remaining increased during all the evaluated time span. *COX1* (Figure 1D) was transiently up-regulated in early periods (1 to 6 h), returning to baseline levels after 12 h. *NCX* (Figure 1E) and *SERCA2* (Figure 1F) were both transiently repressed between 3 and 12 h after application of pressure overload stimuli, returning to their basal expression at 48 h.

Even though we did not evaluate the respective protein levels of the genes studied here, our findings are in agreement with the growing number of studies showing that myocardial hypertrophic response includes temporal variations in the gene program and that most of the transcriptional alterations are translated (Zhao *et al.*, 2004; Spruill *et al.*, 2008; Cullingford *et al.*, 2008). In a later study, the authors observed that most RNAs transcribed in rat cardiomyocytes 1 h following hypertrophic stimuli were equally present in total and polysomal fractions. Hence, changes in RNA expression should be reflected in protein synthesis (Cullingford *et al.*, 2008). Thus, we may suppose that our transcriptional results have protein counterparts playing functional roles in the immediate adjustment of cardiomyocytes to novel force demands.

The switch of myosin isoforms is very well documented as being a response to an increased work load of the myocardium (see review in Gupta, 2007). This is related to cardiomyocyte bioenergetics and their contractile performance due to the lower actomyosin ATPase activity of the beta isoform, thereby leading to an overall decrease in ATP consumption by the overloaded myocardium (Krenz *et al.*, 2007).

**Figure 1:**
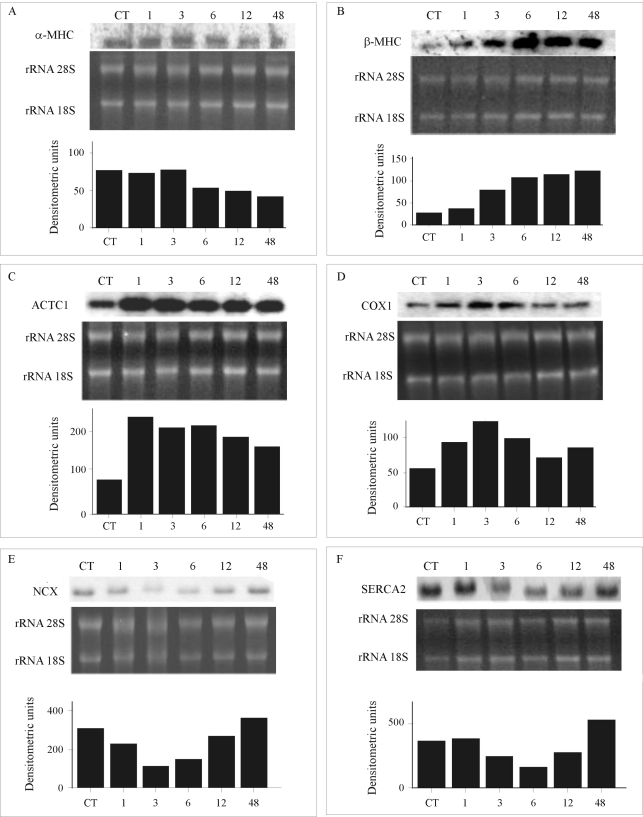
Gene expression followed by Northern blot. A. α*-MHC* (myosin heavy chain, alpha isoform). B. β*-MHC* (myosin, heavy chain, beta isoform). C. *ACTC1* (cardiac actin, alpha). D. *COX1* (cytochrome oxidase subunit I). E. *NCX* (Na^+2^ Ca^+2^ exchanger). F. *SERCA-2* (sarco/endoplasmic reticulum Ca^+2^ ATPase). Control experiments (CT) were obtained with rats that underwent sham surgery. Amount of total RNA used in Northern blot experiments is shown in the middle of each panel.

Also part of the contractile apparatus, the adult isoform of alpha-actin (ACTC1), was immediately up-regulated after PO, maintaining high levels of expression at all the times evaluated herein. This has never been reported before, and is contrary to skeletal alpha actin gene expression which is part of the fetal program (Stilli *et al.*, 2006). We do not fully understand this result, but it is known that the expression of alpha actin isoforms is modulated in the course of hypertrophic heart disease (Machackova *et al.*, 2006; Berni *et al.*, 2009). Thus it is possible that our findings constitute an initial event in this modulatory response, before the other isoform surpasses its expression.

The most significant outcome was the observation of transient expression of genes encoding mitochondrial COX1 and two calcium pumps, SERCA2 and NCX, which occurred during the first hours after PO. Transient up-regulation of the COX1 gene may indicate an adjustment of respiratory fluxes through the mitochondrial electron transport chain in order to attend the higher myocardial energy demands (Goffart *et al.*, 2004). On the other hand, transient down-regulation of genes encoding the two most important mechanisms of cytosolic calcium removal, SERCA2 and NCX, indicates an increase in intracellular calcium levels, which may have multiple consequences on the heart, these including electrophysiology, contractility and signaling.

Interestingly, the transient down-regulation of *SERCA2* and *NCX* may also indicate an immediate adaptation to novel energy requirements in contractile units. Since the beta isoform of myosin has a lower affinity for calcium than the regular adult isoform (Krenz *et al.*, 2007), this possibly indicates higher cytosolic [Ca^+2^] requirements to achieve maximum performance.

Moreover, since both mitochondrial function and intracellular calcium levels are pivotal to several cardiac signaling processes (Wilkins and Molkentin, 2004; Rimbaud *et al.*, 2009), these results may not only constitute an immediate response to PO but also be part of signaling itself. Therefore, our results might reflect the initial short-term alterations that lead to the posterior adjustments of myocardial physiology to the increased work load.

Summarizing, our findings demonstrate that the myocardial gene program is activated in response to an increased work load imposed by aortic constriction. This adaptive response starts quickly during the first hours after pressure overload, as seen by the switch from alpha to beta isoform of the myosin heavy chain gene and up-regulation of the cardiac isoform of alpha actin. There is a transient increase in he expression of mitochondrial cytochrome c oxidase 1 that parallels a transient decrease in expression of the two most important mechanisms of cytosolic calcium removal, sarco(endo)plasmic reticulum Ca^+2^ ATPase and sodium-calcium exchanger genes. Taken together, these results indicate that the first myocardial response to an increased work-load includes the transitory adjustment of mitochondrial bioenergetics and calcium availability, thereby starting physiological adaptations of the myocardium to novel force and energy demands.

## Supplementary Material

The following online material is available for this article:

Detailed Methodology

This material is made available as part of the online article from http://www.scielo.br.gmb.

## Figures and Tables

**Table 1:** Effect of transverse aortic constriction (TAC) on the left ventricular weight (LV) / body weight (BW) ratio and the systolic pressure gradient (SPG). Sham surgery values for LV/BW ratio are shown as control. Values are expressed as mean ± sd; n = 3 for each group.

	1 h	3 h	6 h	12 h	48 h
LV/BW (mg/g) - Sham	2.31 ± 0.27	2.23 ± 0.04	2.12 ± 0.17	2.13 ± 0.10	2.21 ± 0.14
LV/BW (mg/g) - TAC	2.20 ± 0.07	1.86 ± 0.12	2.14 ± 0.01	2.18 ± 0.02	2.52 ± 0.04
SPG (mmHg)	44.0 ± 1.0	45.0 ± 3.0	55.0 ± 1.5	47.0 ± 5.7	52.0 ± 4.0
