# Supplementary material for: Immediate response of myocardium to pressure overload includes transient regulation of genes associated with mitochondrial bioenergetics and calcium availability
Source: Genet Mol Biol. 2010 Mar 1;33(1):12–6. doi: 10.1590/S1415-47572010005000004 (PMC3036092; doi:10.1590/S1415-47572010005000004)
Supplement: Supplementary file 1 — Detailed Methodology [file gmb-33-1-12-suppl1.pdf]

Detailed Methodology

Methods

Animals. Male Wistar rats (160 to 200 g), obtained from animal facilities at the university (UNICAMP, Campinas, Brazil) were used in the experiments. All animals received care in compliance with the principles of laboratory animal care formulated by the UNICAMP Animal Care and Use Committee.

Transverse aortic constriction (TAC). Rats were anesthetized with ketamine (100 mg/kg body weight) and xylazine (5 mg/kg body weight), and placed on a temperature-controlled surgical table. Catheters were placed in the right common carotid artery and right femoral artery for blood pressure measurement. The transverse aorta was dissected and a silver clip (500 um ID) was positioned around the transverse thoracic aorta between the brachiocephalic truncus and the left common carotid branches, in order to induce acute pressure overload. Sham-operated animals underwent an identical procedure except for placement of the clip.

Experimental groups. In periods of 1, 3, 6, 12 and 48 h following TAC (n = 3) or sham surgery (n = 3), rats were euthanized, their hearts extirpated and left ventricles (LV) snap frozen in liquid nitrogen for RNA extraction.

RNA preparation. Samples from each group were pooled together before RNA purification. LVs were powdered with a pestle and mortar in liquid nitrogen and total RNA extracted using TRIZOL (Invitrogen, USA). Quantity and quality were assessed spectrophotometrically and by denaturing agarose gel electrophoresis.

EST library. The 5’ STRETCH PLUS rat heart cDNA Library (Clontech, USA) was used for isolation and purification of clones from phage lysis plates according to the manufacturer’s instructions. DNA amplification was made by PCR in 96-well microplates using the primers λgt11 LD-Insert Screening Amplimers (Clontech, USA).

Sequencing. DNA was sequenced by the Sanger dideoxy method on an ABI PRISM 377 (Perkin Elmer, USA), and quality evaluated with Phred. Similarity searches were performed using BLAST against the GeneBank.

Northern blot. Total RNA (15 μg) was separated according to size using denaturing agarose gel electrophoresis and transferred onto a nylon membrane (Hybond-N+) (GE Healthcare, USA). Radioactive labeled probes were synthesized by incorporation of $[^{32}P]\alpha$-ATP (GE Healthcare, USA) during synthesis of complementary strands (Klenow polymerase) of DNA templates (ESTs of interest). Hybridization was performed at 42 °C for 16 h. After washing (as in microarrays), membranes were autoradiographed. Signals were analyzed after densitometry and normalized using the 18S rRNA.
